# Female-Biased Dispersal and Gene Flow in a Behaviorally Monogamous Mammal, the Large Treeshrew (*Tupaia tana*)

**DOI:** 10.1371/journal.pone.0003228

**Published:** 2008-09-17

**Authors:** Jason Munshi-South

**Affiliations:** Behavior, Ecology, Evolution and Systematics Program, Department of Biology, University of Maryland, College Park, Maryland, United States of America; University of Utah, United States of America

## Abstract

**Background:**

Female-biased dispersal (FBD) is predicted to occur in monogamous species due to local resource competition among females, but evidence for this association in mammals is scarce. The predicted relationship between FBD and monogamy may also be too simplistic, given that many pair-living mammals exhibit substantial extra-pair paternity.

**Methodology/Principal Findings:**

I examined whether dispersal and gene flow are female-biased in the large treeshrew (*Tupaia tana*) in Borneo, a behaviorally monogamous species with a genetic mating system characterized by high rates (50%) of extra-pair paternity. Genetic analyses provided evidence of FBD in this species. As predicted for FBD, I found lower mean values for the corrected assignment index for adult females than for males using seven microsatellite loci, indicating that female individuals were more likely to be immigrants. Adult female pairs were also less related than adult male pairs. Furthermore, comparison of Bayesian coalescent-based estimates of migration rates using maternally and bi-parentally inherited genetic markers suggested that gene flow is female-biased in *T. tana*. The effective number of migrants between populations estimated from mitochondrial DNA sequence was three times higher than the number estimated using autosomal microsatellites.

**Conclusions/Significance:**

These results provide the first evidence of FBD in a behaviorally monogamous species without mating fidelity. I argue that competition among females for feeding territories creates a sexual asymmetry in the costs and benefits of dispersal in treeshrews.

## Introduction

Dispersal exerts an important influence on population genetics and demography, as well as on our ability to predict population-level responses to environmental disturbance [1], [2]. Many vertebrates exhibit sex-biased dispersal, but the pattern differs among taxa: female-biased dispersal (FBD) is typical among birds, whereas males disperse and females are philopatric in most mammals [3]–[5]. Evolutionary models of sex-biased dispersal have drawn comparative support from the prevalence of different mating systems in mammals and birds. Over 90% of bird species live in male-female pairs [behavioral monogamy, 6], whereas 95% or more of mammal species exhibit polygynous mating systems [7]. Theoretical approaches suggest that the same sexual asymmetries driving the evolution of mating systems should also influence the evolution of dispersal patterns [8].

Three non-mutually exclusive factors have been proposed to explain the association between mating systems and sex-biased dispersal: inbreeding avoidance, local resource competition (LRC), and local mate competition [reviewed in 9]. All three hypotheses predict male-biased dispersal in polygynous species, because male offspring may be more likely to mate with the care-giving parent (i.e. females often have longer tenure), face more intense local competition for mates, or compete for resources to attract females, respectively. Sexual asymmetries in mate competition and risk of inbreeding are not predicted under monogamy, because individuals of both sexes may have only one mate and the same number of offspring. However, intense local resource competition may lead to FBD in monogamous species when dispersing females gain critical resources for reproduction [3].

Monogamy in mammals is associated with female use of exclusive territories [10], primarily as a strategy to minimize feeding competition when predation and other factors do not favor group-living [11]–[13]. Reproduction in males is unlikely to be as severely limited by food resources as it is in females, and thus an asymmetry in the costs of philopatry may arise in monogamous species if females compete for access to feeding territories. LRC may also increase the rate of female aggression in multi-female groups, resulting in the expulsion of juvenile females by their mothers [e.g. primates 14], [15]. However, comparative data suggest that most juvenile dispersal is “voluntary” [16], because the costs of dispersal may be low when unoccupied areas are available to immigrants [17].

The predicted association between FBD and monogamy has rarely been examined in mammals, largely because most mammals are polygynous [7]. Dobson's [4] comparative study did not find an association between FBD and monogamy in mammals, but few data were (and still are) available for monogamous species. Lawson Handley [18] found evidence for FBD in only four monogamous mammals, although other cases may exist [e.g. 19]. Unbiased measures of dispersal are difficult to obtain using traditional techniques, especially for pair-living species that are widely dispersed in space and time. Sex biases in dispersal may also be obscured by the geographic scale at which a given study is conducted [20], [21]. However, genetic methods to detect both sex-biased dispersal and gene flow at varying spatial scales have recently become available that ameliorate these logistical problems [22], [23].

Several polygynous mammals have been studied using these genetic techniques, and as predicted either no sex bias [e.g. river otters, *Lontra canadensis*, 24] or male-biased dispersal [e.g. brush]-[tailed rock wallabies, *Petrogale penicillata*, 25, talar tuco–tucos, *Ctenomys talarum*, 26] has been detected in most cases. However, genetic analyses have revealed FBD multiple times in polygynous species [common wombats], [ *Vombatus ursinus*], [ 27], [bush hyraxes, *Heterohyrax brucei*, 28, kinkajous, *Potos flavus*, 29, greater white–lined bats, *Saccopteryx bilineata*, 30], especially among catarrhine primates [chimpanzees], [*Pan troglodytes*], [31], [humans], [*Homo sapiens*], [32, western gorillas, *Gorilla beringei*, 33,34, bonobos, *Pan paniscus*, 35, hamadryas baboons, *Papio hamadryas*, 36]. Genetic studies conducted on behaviorally monogamous mammals have found no evidence of sex-biased dispersal [fat–tailed dwarf lemurs, *Cheirogaleus medius*, 37], or contrasting patterns. Male alpine marmots (*Marmota marmota*) disperse more often than females [38], whereas FBD occurs in the greater white-toothed shrew [*Crocidura russula*, 9]. In a review of sex-biased dispersal in mammals, FBD did not strongly correlate with any particular mating system but was found in taxonomic clusters (e.g. Atelidae). Additionally, some studies have detected FBD in polygynous species where local mate competition is known to be intense [Table 2 in 18].

Predictions of a simple association between FBD and monogamy are complicated by increasingly common genetic results indicating substantial extra-pair paternity in putatively monogamous mammals [e.g. 52% in swift foxes], [*Vulpes velox*, 39, 35% in mountain brushtail possums, *Trichosurus cunninghami*, 40]. Although these species may live in male-female pairs that occupy joint territories (i.e. behavioral monogamy), the genetic mating system more closely resembles polygyny where individual males sire offspring with multiple females. Thus, male-biased dispersal due to inbreeding avoidance, LRC or local mate competition between males may occur in these species. Alternatively, LRC among females may be intense enough to favor FBD despite extra-pair paternity comprising an important component of the genetic mating system. FBD is prevalent among birds even though most avian species studied to date exhibit behavioral monogamy and extra-pair paternity in greater than 5% of offspring [112/130 bird species reviewed in 41]. These results are likely due to the benefits males gain from philopatry by defending a successful breeding territory to attract females. Few tests of sex-biased dispersal have been conducted in behaviorally monogamous mammals without mating fidelity. Alpine marmots exhibit moderate extra-pair paternity [19%, 42] and male-biased dispersal, whereas fat-tailed dwarf lemurs exhibit substantial extra-pair paternity [44%, 43] but no sex bias in dispersal. Additional case studies are clearly needed to determine whether FBD occurs in pair-living mammals with or without extra-pair paternity.

In this study I use multiple genetic methods to test for FBD and gene flow in the large treeshrew (*Tupaia tana*) in NE Borneo. Large treeshrews form behaviorally monogamous pairs that forage solitarily, potentially as an adaptation to intraspecific foraging competition [dispersed pairs], [12,44]. The rate of extra-pair paternity in *T. tana* is one of the highest ever recorded for a mammal (50%), but variance in reproductive success does not vary between males and females [45]. Comparative analysis of testis size also indicates that male *T. tana* are not subject to intense sperm competition [45], and thus competition among males for mates or resources may not be strong enough to favor male-biased dispersal. Alternatively, FBD may occur in this species due to unique energetic limitations that produce intense competition between females. Treeshrews exhibit an absentee maternal care system that preempts reproduction when resources are scarce [46]. Females deposit their two young in a nest chamber and visit them only once every 48 hours for intensive nursing. In the interim, females devote most of their activity period to foraging [10.5 hrs daily, 46] and travel long daily distances (means = 1.1–1.5 km depending on year and study site) for their body size [means = 202–257 g, 44] to produce and store the required large amounts of milk. Females exhibit sex-specific territorial defense [46], and competition among females for feeding territories to support their physiologically expensive foraging and maternal behavior may result in FBD.

I tested the prediction of FBD in *T. tana* by comparing the genetic structure and patterns of relatedness among adult males and females at seven autosomal microsatellite loci. FBD is predicted to produce genotypes with lower population assignment probabilities and pairwise relatedness among adult (i.e. post-natal dispersal) females than among adult males in the population [22]. I also examined the prediction of female-biased gene flow in *T. tana* by comparing gene flow estimated from bi-parentally inherited microsatellite markers and a maternally inherited mitochondrial DNA (mtDNA) marker. Bayesian methods based on the coalescent [47] were used to estimate the exchange of migrants between two different *T. tana* populations. If gene flow is female-biased, then the migration rate for mtDNA should substantially exceed the migration rate for bi-parentally inherited microsatellites.

## Materials and Methods

### Study sites and genetic sampling

Large treeshrews are small (200–250 g), diurnal, frugivore-insectivores that inhabit the lowland tropical rainforests of Borneo and Sumatra. I collected ear clips for genetic analyses from 54 *T. tana* individuals at two sites in Sabah, Malaysia (NE Borneo) from 2002–2004 during a larger study on mating systems in treeshrews. The first site (*N* = 39 samples) was located in the Danum Valley Conservation Area (Danum, 4°58′N, 117°48′E) and consisted of undisturbed primary lowland rainforest. The other site (*N = *15 samples) was located 53 km away in the Malua Forest Reserve (5°5′N, 117°38′E). This area was heavily logged in the early 1980's and has yet to recover the multiple closed canopies (typically 10 m and 20–30 m in height) and tall emergent trees (up to 70 m) that characterize lowland rainforests in SE Asia [48]. See Munshi-South *et al.* [44] for full details of the study sites and trapping methods. This research was approved by the Institutional Animal Care and Use Committee at the University of Maryland, and adhered to all laws governing research in USA and Malaysia.

I extracted genomic DNA from ear tissue samples using Qiagen DNEasy tissue extraction kits (Qiagen, Valencia, CA). Seven previously-described, unlinked microsatellite loci named JS22, JS132, JS183, JS188, JS196, SKTg19, and SKTg22 were amplified from DNA extracts using the PCR conditions in Munshi-South & Wilkinson [49]. Fluorescently-labeled alleles were separated on an Applied Biosystems 3100 DNA Analyzer and sized and scored using Genotyper 2.5 (Applied Biosystems, Foster City, CA). I also PCR-amplified a 602 bp segment of the mtDNA control region with the primers JMSTbel386 and JMSTbel1110 and PCR conditions described in Munshi-South [45]. PCR products were sequenced using the BigDye Terminator 3.1 and an ABI 3100 DNA Analyzer, and then sequences were edited and aligned using Sequencer 4.1.2 (Gene Codes, Ann Arbor, MI) and Bioedit 7.0.4.1 [50].

To examine differences in genetic variability between the primary and logged forest populations, I calculated the number of alleles and allelic richness at each microsatellite locus for each population using FSTAT v. 2.9.3.2 [51]. I also used the log-likelihood G test of genotypic differentiation implemented in FSTAT [10,000 randomizations not assuming Hardy–Weinberg equilibrium, 52] to examine whether the two populations exhibited significantly different microsatellite allele frequencies. I investigated mtDNA sequence divergence between populations by calculating the number of fixed differences and shared mutations between populations, and the average nucleotide substitutions and number of net substitutions per site between populations [*D_xy_* and *D_a_*, respectively, with Jukes–Cantor correction, 53], using DNASP v. 4.2.4 [54]. I also conducted a permutation test (10,000 randomizations without alignment gaps) of genetic differentiation using the nearest-neighbor statistic (*S*_nn_) implemented in DNASP. *S*_nn_ measures how often the most similar sequences in a data set (“nearest neighbors”) are from the same population, and produces a powerful test of genetic differentiation for sequence data in nearly all situations [55].

### Tests of female-biased dispersal

To test for FBD, I compared mean corrected assignment indices (*mAI*_c_) between adult males and females using the “biased dispersal” module in FSTAT. One-sided *P* values were calculated using 10,000 randomizations. The assignment index is the probability that an individual's genotype occurred by chance in a population [56], and Favre et al. [9] applied a correction that produces mean *AI_c_* values of zero for each population. Negative *AI_c_* values characterize individuals with genotypes less likely than average to occur in a population sample, and thus lower *mAI*_c_ values for one sex (females, in this case) implies sex-biased dispersal. This index was chosen because both simulations and real data sets have indicated that this test has high power at detecting moderately intense biases in dispersal [22], [57]. Adult genotypes were used for these analyses, because this test assumes post-dispersal sampling (*N* = 14 females and 20 males).

I also tested the prediction that pairs of adult females were less related on average than pairs of adult males, because sex-biased dispersal is predicted to influence local relatedness structure among adults [e.g. 25], [27], [58]. If female *T. tana* disperse more often or farther than males, then fewer closely related pairs of females should occur in the sample. I calculated two estimates of pairwise relatedness, because the performance of different estimators varies depending on population composition [59]. Two method-of-moment regression estimators, Lynch and Ritland's *r* [60] and Queller and Goodnight's *r* [61], were calculated using the program MARK [62]. Simulations indicate that the Lynch and Ritland estimator performs well for most population compositions [63]. The Queller and Goodnight estimator is commonly used in studies of relatedness, and was included to facilitate comparison with other studies.

Pairwise relatedness estimates from the primary and logged forest populations were pooled to increase sample sizes, but relatedness was calculated only between pairs of individuals from the same population. Using only dyads from the same population gives a better representation of background population-level allele frequencies. For each different estimator, I tested whether mean female relatedness was lower than male relatedness using a two-sample randomization test [64]. Randomization tests were used because relatedness data were generated for dyads of individuals and thus do not represent independent observations. The one-sided *P* value for these tests was calculated by comparing the observed mean difference to the mean differences calculated from 10,000 randomizations of the same sets of relatedness estimates using POPTOOLS 2.6 [65].

### Tests of female-biased gene flow

If gene flow among large treeshrews is female-biased, then migration rates calculated for maternally inherited mtDNA should be higher than migration rates calculated for bi-parentally inherited autosomal markers. To test this prediction, I used the Bayesian coalescence approach implemented in MIGRATE 2.1.3 [66] to estimate the effective number of migrants exchanged per generation (*N_e_m*) between the two populations using both the microsatellite and mtDNA sequence data. Bayesian inference may be more accurate and efficient at sampling genealogy space than maximum likelihood approaches for many datasets [47]. This method produces estimates of Θ (4*N_e_μ*, where *μ* = mutation rate) and *M* (*m*/*μ*) from microsatellite data, equaling 4*N_e_m* when multiplied together. For mtDNA, this method estimates 2*N_f_m* (*N_f_*  = effective population size of females). Assuming an equal sex ratio and equal variance in reproductive success among males and females, *N_f_* is equivalent to *N_e_*/2 calculated from microsatellites. Higher migration rates for mtDNA than for microsatellites should thus indicate female-biased gene flow.

To estimate the effective number of migrants from microsatellite data, I ran 10 sequential iterations in MIGRATE using a stepwise mutation model with constant mutation rates, an exponential prior distribution (Θ distribution: minimum = 0.0, maximum = 0.1, mean = 0.01; *M* distribution: minimum = 0.000001, maximum = 1000, mean = 100), starting parameters based on *F_st_* calculations, burn-in equaling 10,000 trees, five long chains sampling 2,000,000 genealogies, and an adaptive heating scheme (swapping interval = 1; four chains with start temperatures = 1, 1.2, 1.5 and 3). The same analysis was then repeated using the estimates of Θ and *M* obtained from the first analysis as starting parameters. In this second analysis, a search window for the exponential prior distribution was set according to the distribution of parameter estimates from the first analysis (Δ = 0.03 for Θ; Δ = 110 for *M*). For the mtDNA dataset, I used the same analytical strategy with the F84 model of DNA sequence evolution instead of the stepwise microsatellite mutation model. However, I increased the number of sampled genealogies to 10,000,000 to achieve convergence, and used wider windows in the second run (Δ = 0.06 for Θ; Δ = 250 for *M*). These analyses produced values of Θ*M* (4*N_e_m* and 2*N_f_m* for microsatellites and mtDNA, respectively) estimated in each direction between the two populations along with their approximate 95% confidence intervals [0.025 and 0.975 posterior distribution values, 66]. Following Wright et al. [67], I then calculated the overall number of migrants per generation (*N_e_m*) by summing Θ*M* in each direction and dividing by four for microsatellites and two for mtDNA.

## Results

### Genetic differentiation between primary and logged forest populations

Microsatellite allelic diversity was moderate in both *T. tana* populations, ranging from two to nine alleles (mean = 6.43) in the primary forest and from two to six alleles (mean = 4.0) in the logged forest (Table 1). Allelic richness, a measure of allelic diversity independent of sample size, showed a similar pattern (Table 1). Genotypic differentiation between the two populations was highly significant overall (*P*<0.0001), as well as for four out of the seven loci (JS183, JS188, SKTg19, and SKTg22; Table 1). There were zero fixed differences and 14 shared mutations between populations in the 602 bp mtDNA d-loop sequence. The average number of nucleotide substitutions per site between populations was *D*_xy_±SD = 0.026±0.007, and the net substitutions per site was *D*_a_±SD = 0.0016±0.006. In contrast to the microsatellite genotypes, genetic differentiation in the mtDNA sequence was not significant between the two populations (*S*_nn_ = 0.66, *P* = 0.16).

**Table 1:** Number of alleles and allelic richness of seven microsatellite loci among large treeshrews from the primary forest (*N* = 39) and logged forest (*N* = 15) populations.

	No. alleles			Allelic richness			
Locus	Primary	Logged	Total	Primary	Logged	Total	*P* value
JS22	9	5	10	6.33	4.87	6.18	0.11
JS132	2	2	2	2	2	2	0.64
JS183	12	6	12	8.74	5.93	8.68	0.02
JS188	6	6	8	4.86	5.93	5.86	<0.001
JS196	4	3	4	3.76	3.0	3.57	0.55
SKTg19	6	2	6	4.48	2.0	4.08	0.03
SKTg22	6	4	7	5.79	4	6.31	<0.0001
Mean	6.43	4.0	7.0	5.14	3.96	5.24	

*P* values correspond to 10,000 randomizations of log-likelihood *G* tests of population differentiation for each locus. The test of population differentiation over all loci was highly significant (*P*<0.0001).

### Female-biased dispersal

**Figure 1:**
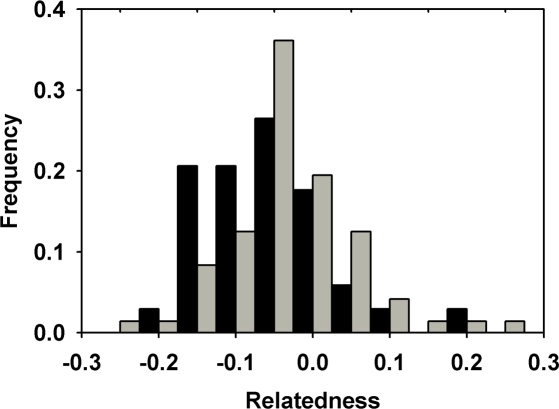
Frequencies of pairwise relatedness values (Lynch & Ritland's *r*) for male (gray bars) and female (black bars) large treeshrews.

In agreement with predictions for FBD, I found significantly lower *mAI*_c_ for adult females than for adult males (Table 2). Mean *AI*_c_ was negative for females (mean = −0.70) and positive for males (mean = 0.48), indicating that females are more likely to be immigrants than males. Two method-of-moment estimators of relatedness, Lynch and Ritland's *r* (Figure 1) and Queller and Goodnight's *r*, also indicated that adult female pairs were significantly less related than adult males (*P*<0.05; Table 2).

**Table 2:** Mean values and tests of FBD based on the corrected assignment index (*mAI*_c_) and two relatedness estimators.

Test	Male	Female	*P*
*mAI*_c_	0.48	−0.70	<0.05
Lynch-Ritland *r*	−0.05	−0.09	<0.05
Queller-Goodnight *r*	−0.04	−0.09	<0.05

### Female-biased gene flow

Bayesian inference of migration rates produced an estimate for mtDNA of 2*N_f_m* = 8.20 (95^th^ percentile = 1.64–24.56) from primary to logged forest and 2*N_f_m* = 3.35 (95^th^ percentile = 0.07–13.46) from logged to primary forest. These two estimates produce an overall estimate of *N_f_m* = 5.77. Assuming an equal sex ratio and low variance in male reproductive success, this value is equivalent to *N_e_m* = 11.54 effective migrants exchanged per generation between the two populations.

Microsatellite estimates of the effective number of migrants were substantially less than mtDNA estimates. Bayesian inference produced an estimate across all seven loci of 4*N_e_m* = 12.26 (95^th^ percentile = 5.93–15.27) from primary to logged forest and 4*N_e_m* = 2.04 (95^th^ percentile = 1.05–3.40) from logged to primary forest. These estimates correspond to an overall effective number of migrants exchanged per generation of *N_e_m* = 3.58, which is more than three times less than *N_e_m* estimated for mtDNA.

## Discussion

Multiple genetic analyses presented here provide evidence of FBD in large treeshrews. As predicted for FBD, adult females had significantly lower mean values than males for two different tests (*mAI_c_* and pairwise relatedness). These methods detect sex-biased dispersal only when adults have been thoroughly sampled and the sex bias is intense [e.g. 80:20 in simulated datasets, 22]. A sex bias was detected for *T. tana* despite moderate sample sizes and genetic variability at seven microsatellite markers, suggesting that dispersal is substantially female-biased in this species. The magnitude of the difference between males and females in *mAI*_c_ (1.18) for large treeshrews was similar to values for two other cases where a sex bias was also confirmed using trapping data [mean of 1.82 in white]-[toothed shrews, 9, 1.35 in white–footed mice, 57].

Evidence of FBD in *T. tana* was also provided by significantly lower relatedness values among adult females than among males for two pairwise measures of relatedness. Average male and female relatedness were negative for two method-of-moment regression estimators (Figure 1), but negative relatedness values are not unexpected given the high sampling variance of these estimators inherent in all but the largest data sets [60], [e.g. >40 loci, 63]. Negative pairwise relatedness results whenever one pair member exhibits the other's alleles at a frequency less than the estimated population frequency [68]. Female relatedness may thus be negative more often if immigrant females with genotypes that do not reflect overall population allele frequencies are present in the sample. A relatively large proportion of related individuals (e.g. male relatives, as predicted if males disperse less often) in the sample could also contribute to negative relatedness for unrelated females. These methods do not distinguish between biases in the numbers of individuals of each sex dispersing vs. the distances dispersed. This study also did not address whether males are actually philopatric, but male offspring born in one study period were typically not present on their natal territory in the following study period [45]. The differences in *mAI_c_* and relatedness for *T. tana* were likely caused by females with uncommon genotypes that immigrated to the study site (i.e. a bias in the dispersal distance) rather than male philopatry.

The prediction of greater migration (i.e. gene flow) rates for maternally inherited markers than bi-parentally inherited markers was also supported. The overall number of migrants per generation estimated using mtDNA was more than three times higher than the microsatellite estimate. The substantially higher migration rate for mtDNA suggests that historical gene flow in large treeshrews has been female-biased. Recent studies have raised concerns that migration rates and confidence intervals estimated from mtDNA using maximum likelihood coalescence techniques are often not accurate [69]. However, the Bayesian coalescence approach implemented in this study ameliorates these problems by achieving improved accuracy and more thorough genealogical sampling [47]. The magnitude of the difference in migration for mtDNA and microsatellite markers may be reduced if *T. tana* samples for this study violate the assumptions of an equal sex ratio and equal variance in male and female reproductive success. However, variance in reproductive success was not different between males and females, and the sex ratio of offspring was equal in these populations [45], indicating that these assumptions are reasonable for *T. tana*.

This study is only the second to find genetic evidence of FBD, and the first to report female-biased gene flow, in a behaviorally monogamous mammal. Evidence for FBD is more prevalent among polygynous mammals [especially primates], [e.g. 27,36] with social and mating systems characterized by inbreeding avoidance and male kin-cooperation rather than LRC [19 spp., 18]. The only other genetic evidence of FBD in a behaviorally monogamous species comes from studies on a temperate shrew *C. russula*, which also exhibited lower *mAI_c_* values among females than males [9]. However, behavioral pairs of *C. russula* only persist for less than one breeding season, placing them at the short-term end of the continuum of pair duration in behaviorally monogamous mammals [13]. Large treeshrews represent a unique case study of FBD because they form behaviorally monogamous pairs that persist for several breeding periods [and potentially for life, 46], but also exhibit substantial extra-pair paternity [45]. Thus, one might predict the opposite sex bias in dispersal due to competition between male treeshrews for extra-pair copulations. Potentially unexpected results such as these from treeshrews highlight the need to identify specific pressures driving FBD in species with contrasting mating systems [18].

Greenwood [3] predicted that monogamy would correlate with FBD because a sexual asymmetry in the costs of resource competition may favor the evolution of these two behavioral patterns. Foraging competition is the most likely driver of the evolution of behavioral monogamy in large treeshrews [44], and would also be expected to exert evolutionary pressure on dispersal patterns. Treeshrews live in behaviorally monogamous pairs, but forage solitarily and do not share sleeping sites. This dispersed form of behavioral monogamy likely arose through a two-step evolutionary scenario: female avoidance and territoriality due to foraging competition, followed by male defense of a single female's territory to prevent other males feeding in the same area [intersexual feeding competition hypothesis], [44,70]. Female body condition and reproductive output increase during supra-annual fruit masting events in Borneo, suggesting that fruit abundance is a key factor limiting reproduction in this species [44], [46]. The unique, energetically-expensive absentee maternal care system of *T. tana* may also limit the ability of females to produce young on poor-quality territories, or during periods of resource scarcity. These physiological and behavioral limitations on reproduction are likely to produce intense competition between females for resources, and may be the main factor driving females to disperse away from their natal territory to settle on a high-quality territory for their own reproduction.

The costs and benefits influencing the evolution of behavioral monogamy appear to influence dispersal patterns in large treeshrews. The fitness benefits that females gain from dispersal and the proximate factors influencing dispersal rates are fruitful areas for future research that could be addressed using provisioning experiments. Benefits males gain from philopatry, if any and if they are indeed philopatric, also deserve closer examination. The results from this study also indicate that gene flow is ongoing between *T. tana* populations in primary forests and logged forests in Sabah, Malaysia. Southeast Asia has experienced greater rates of deforestation than other tropical regions [71], and Sabah is typical in that most of the valuable timber has already been extracted from its lowland rainforests [72]. Most vertebrate species are present after logging, but the connectivity of populations in primary and logged forests is not well understood [73]. I found significant genotypic differentiation at microsatellite loci between the primary and logged forest populations, but gene flow estimated for mtDNA suggests that female migration may be sufficiently high to avoid rapid loss of genetic variation among large treeshrews in Sabah.
